# Correlation of axial length and myopic macular degeneration to levels of molecular factors in the aqueous

**DOI:** 10.1038/s41598-019-52156-y

**Published:** 2019-10-31

**Authors:** Chee Wai Wong, Yasuo Yanagi, Andrew Shih Hsiang Tsai, Waseem Ahamed Shihabuddeen, Ning Cheung, Shu Yen Lee, Jost B. Jonas, Chui Ming Gemmy Cheung

**Affiliations:** 1 Singapore Eye Research Institute, Singapore National Eye Centre, Singapore, Singapore; 2 Ophthalmology and Visual Sciences Academic Clinical Program, Duke-NUS Medical School, National University of Singapore, Singapore, Singapore; 3 Department of Ophthalmology, Medical Faculty Mannheim of the Ruprecht-Karls-University, Mannheim, Germany

**Keywords:** Refractive errors, Retinal diseases

## Abstract

To elucidate the molecular processes associated with the development of myopic macular degeneration (MMD), we measured the intraocular concentrations of molecular factors in emmetropic and myopic eyes. This is a retrospective clinic-based case-control study that included eyes undergoing routine cataract surgery whereby aqueous humour samples were obtained. We measured the concentrations of pigment epithelium derived factor(PEDF), matrix metalloproteinase 2(MMP-2), tissue inhibitor of metalloproteinase(TIMP-2), vascular endothelial growth factor isoform A(VEGF-A), interleukin 8(IL-8), interleukin 6(IL-6), C-reactive protein(CRP), angiopoietin 2(Ang2), and amphiregulin. 38 eyes (axial length (AL): 22.4–32.4 mm), including 12 highly myopic (HM) eyes (AL ≥ 26.5 mm) without MMD and 12 HM eyes with MMD but without neovascularization were included. Eyes with MMD were found to have significantly lower VEGF-A levels (p = 0.007) and higher MMP-2 levels (p = 0.02) than control eyes after adjusting for age and gender. MMP-2 levels correlated positively (r = 0.58, p = 0.002), while VEGF-A levels correlated negatively with longer axial length (r = −0.75, p < 0.001). Both the concentrations of VEGF-A (*P* = 0.25) and MMP-2 (*P* = 0.69) were not significantly associated with MMD after adjusting for AL. These findings suggest that the predominant mechanism underlying the development of non-neovascular MMD may be axial elongation, driven in part by MMP-2 related mechanisms.

## Introduction

The prevalence of myopia and high myopia is increasing markedly worldwide, and the sequelae such as myopic choroidal neovascularization and myopic macular degeneration (MMD) are emerging as leading causes of irreversible blindness worldwide^1,2^. Few studies have assessed so far the molecular pathways involved in the process of the development of myopic macular degeneration (MMD)^3–8^. It has remained unclear whether the development of MMD reflects the severe end of the spectrum of axial myopia as a consequence of extreme axial elongation, or whether specific pathogenic mechanisms are additionally involved.

The target pathways and their related molecular alterations can be broadly grouped as those related to scleral remodeling (matrix metalloproteinase (MMP), tissue inhibitor of metalloproteinase (TIMP)^7^), active Bruch’s membrane elongation^9^ (amphiregulin^10^), inflammation (interleukin 6 (IL-6)^11,12^, and those related to vascular/atrophic changes (pigment epithelium derived factor (PEDF)^3,13^, vascular endothelial growth factor (VEGF), angiopoietin (Ang))^3–5,8^. Angiopoietin, VEGF and PEDF are synthesized by retinal pigment epithelial cells and are vital for the maintenance of the choriocapillaris^14^. Low concentrations of these factors may contribute to the choroidal thinning and chorioretinal atrophy seen in MMD. Various inflammatory conditions, both systemic and ocular, have been associated with myopia. A chronic inflammatory state, which may be represented by raised inflammatory concentrations of cytokines including interleukin-6 and interleukin-8, may play a role in MMD^11,12^. Scleral remodeling resulting in abnormal elongation of the globe is closely related to the presence of degenerative changes seen in pathologic myopia, and these changes may be reflected at the molecular level by alterations in the levels of MMP-2 and TIMP-2^15^. Amphiregulin has been postulated to drive axial elongation by stimulating the elongation of Bruch’s membrane in the equatorial and retro-equatorial region. The increase in the length of Bruch’s membrane in the midperiphery has been hypothesized to push Bruch’s membrane at the posterior pole backward, leading to a compression and thinning of the choroid and to a re-modelling of the sclera. These changes might lead to an increase in tension within Bruch’s membrane and eventually to macular defects in Bruch’s membrane as part of myopic macular degeneration^9^.

While studies have compared the aqueous humor levels of these molecular factors in highly myopic eyes with non-myopic controls^3,4,8,13,16–18^, none of these studies have examined the relationship of these molecular factors specific to MMD. We sought to gain a better understanding of these molecular mechanisms underlying the process of axial elongation and the development of high myopia and MMD. The purpose of this study was to assess and compare the aqueous concentrations of MMP-2, TIMP-2, PEDF, VEGF-A, Ang2, IL-6, IL-8, C-reactive protein (CRP) and amphiregulin in non-highly myopic eyes and highly myopic eyes without and with MMD.

## Methods

This was a retrospective clinic-based case control study of eyes with high myopia (HM), defined as axial length ≥26.5 mm, and a control group of non-highly myopic eyes with no diabetes mellitus and no other ocular pathology other than age related cataract. Patients were recruited before cataract surgery from the outpatient clinics of the Singapore National Eye Centre from January 2015 to December 2017, and informed consent was obtained. Exclusion criteria were diabetes mellitus and any optic nerve disease and retinal disorder other than myopic maculopathy. Eyes with active, scarred or atrophic myopic choroidal neovascularization were also excluded. Axial length was measured by partial coherence interferometry (IOL Master, Carl Zeiss Meditec AG, Jena, Germany). The study was approved by the SingHealth Centralized Institutional Review Board (protocol number R1256/62/2015) and conducted in accordance with the principles of the Declaration of Helsinki.

The grading of myopic macular degeneration was performed by examining 45° color fundus photographs obtained pre-operatively by a digital fundus camera (Canon CR-DGi with Canon EOS 10D SLR backing; Canon Inc, Tokyo, Japan) after pupillary dilation with tropicamide (1%) and phenylephrine (2.5%). A single fundus field of the eye was photographed, centered on the fovea. Two fellowship trained retinal specialists (CWW, AT) masked to the participant characteristics graded the fundus photographs using the META-PM classification system^19^. Significant MMD was defined as META-PM category ≥2. Spectral domain optical coherence tomography (SD-OCT) with enhanced depth imaging (EDI) was performed pre-operatively (Spectralis HRA-OCT; Heidelberg Engineering, Heidelberg, Germany). Subfoveal choroidal thickness was measured manually at the fovea with the built-in calipers of the viewing software as the distance from the outer surface of the hyper-reflective line representing the retinal pigment epithelium, to the hyper-reflective line of the choroid-scleral interface^20^. All choroidal thickness measurements were performed by two fellowship trained retinal specialists (CWW, AT) who were masked to patient characteristics.

All study participants underwent routine cataract surgery, and the aqueous samples were collected at the beginning of surgery. A paralimbal paracentesis was routinely performed as part of the usual cataract surgery procedure. A 31-gauge needle on a 3 ml syringe was used to aspirate 0.2 ml of aqueous humour through the paracentesis. The aqueous samples were immediately stored at −80°.

All aqueous samples were analyzed separately. A planar bead-based multiplex immunoassay was used to quantify levels of IL-6, IL-8, CRP, Angiopoeitin-2 and VEGF-A in aqueous humor using the LUNARIS™ Ophthalmology kit (AYOXXA Biosystems GmbH, Cologne, Germany). This new technique followed the sandwich ELISA principle and utilized less than 5 μl of samples per well on a 384-well format biochip. Each well contained different antibody-coated microspheres immobilized on microcavities assigned with specific positional coordinates for bead identification, enabling simultaneous measurements of multiple analytes within each sample. Fluorescence intensity of each bead was measured using the LUNARIS chip reader where the bead signal is proportional to the concentration of each analyte of interest calculated from the calibration curves generated using the LUNARIS Analysis Suite software. The aqueous concentrations of amphiregulin, MMP-2, TIMP-2 and PEDF were determined by conventional ELISA since these factors are not yet available in the multiplex platform. Four different ELISA kits were used: Human Amphiregulin ELISA Kit (Sigma-Aldrich, cat. no. RAB0019-1KT), Human MMP 2 ELISA kit (Sigma Aldrich, cat. No. RAB0365-1KT), TIMP 2 (Sigma-Aldrich, cat. No. RAB0472-1KT), and PEDF (iDNA Biotechnology, cat. No. RD191114200R). The kits were supplied with an antibody pre-coated 96-well plate, biotinylated detection antibody, a horseradish peroxidase linked to streptavidin as enzyme and 3, 3′, 5, 5′-tetramethylbenzidine (TMB) as substrate. Both the multiplex assay and ELISA were performed in accordance with the manufacturer’s specifications. All aqueous samples were diluted two-fold with sample diluents provided in each kit and were assayed in duplicate. The limits of detection were 2.122 pg/ml, 311.488 pg/ml, 252.720 pg/ml and 5.493 pg/ml for amphiregulin, PEDF, MMP-2 and TIMP-2 respectively. Amphiregulin was measured in 31 out of 38 samples (14 controls, 7 HM without MMD, 10 HM with MMD) due to insufficient aqueous humour volume to perform the analysis in 7 samples.

Statistical analysis was performed with Stata 13.0 (Stata Corporation, College Station, TX). A sample size calculation was performed using VEGF-A as the main outcome measure, and a sample size of 12 for each group was needed to achieve a power of 80% and a level of significance of 5%^21^. Normality of distribution was tested with the Shapiro Wilk test. Continuous baseline characteristics and aqueous molecular factor levels were compared with the independent student t-test while proportions were analyzed with the chi-square test. One-way ANOVA (analysis of variance) was performed to the compare molecular factor levels between high myopes with and without MMD and controls. Multiple comparisons were adjusted using Bonferroni´s method to correct for performing multiple comparisons. The *P*-value for trend across ordinal categories (high myopes with and without MMD and controls) were calculated, and multivariable analysis was performed with ordinal logistic regression adjusted for axial length. Correlations between molecular factors, axial length and choroidal thickness were analyzed using Spearman’s correlation coefficient. We calculated the non-standardized regression coefficient beta, the standardized regression coefficient B, and its 95% confidence interval (CI). A two-tailed *P*-value of <0.05 was considered statistically significant.

## Results

The study included 24 highly myopic patients and 14 control patients (Table 1). Within the highly myopic group, MMD was detected in 12 eyes (12/24 or 50%), with 7 eyes showing META-PM category 2 and 5 eyes having META-PM category 3. None of the eyes had any previous choroidal neovascularization nor had been treated for it. None of the eyes with META-PM category 3 MMD were observed to have macular Bruch’s membrane defects on spectral domain OCT. All three groups did not differ significantly (*P* = 0.74) in age which ranged from 43 years to 78 years (Table 1). Mean axial length increased significantly (*P* < 0.001) from the control group (24.3 ± 1.1 mm) to the highly myopic group without MMD (28.1 ± 1.5 mm) and eventually to the highly myopic group with MMD (29.5 ± 1.2 mm). In a parallel manner, the prevalence of females versus males increased significantly (*P trend* = 0.01) (Table 1).

**Table 1:** Patient characteristics.

n	Control group	High myopia without myopic macular degeneration	High myopia with myopic macular degeneration	*P*-trend**	*P*-value^††^
	14	12	12		
Age, years	63.9 ± 6.2	59.1 ± 8.6	63.8 ± 10.1	0.86	0.28
Female Gender (%)	5 (36%)	6 (50%)	11 (91.2%)	0.01	0.01*
Axial Length, mm	24.3 ± 1.1	28.1 ± 1.5	29.5 ± 1.2	<0.001	<0.001^†^
Subfoveal Choroidal thickness, um	215 ± 21	80.3 ± 67.0	51.5 ± 33.3	0.001	<0.001^‡^

*Pairwise comparison with Bonferroni correction: controls vs high myopes without myopic macular degeneration (MMD), p < 0.001; controls vs high myopes with MMD, p < 0.001; high myopes without MMD vs high myopes with MMD, p = 0.002.

^†^Pairwise comparison with Bonferroni correction: controls vs high myopes without MMD, p < 0.001; controls vs high myopes with MMD, p < 0.001; high myopes without MMD vs high myopes with MMD, p = 0.057.

^‡^Pairwise comparison with Bonferroni correction: controls vs high myopes without MMD, p = 0.001; controls vs high myopes with MMD, p < 0.001; high myopes without MMD vs high myopes with MMD, p = 0.90.

**P-trend was analyzed across the ordinal categories of controls, high myopia without MMD and high myopia without MMD. A two-tailed *P*-value of <0.05 was considered statistically significant.

^††^P-value was obtained using one way analysis of covariance (ANOVA) across the 3 groups. A two-tailed *P*-value of <0.05 was considered statistically significant.

Table 2 compares the molecular factor levels in the 3 groups. The amphiregulin concentrations were lower than the limit of detection in 19 eyes (61%), and for the purpose of the statistical analysis, the amphiregulin concentrations in these eyes were assumed to be zero. None of the other molecular factors had measurements below the limit of detection. Eyes with MMD were found to have significantly lower VEGF-A levels (136 ± 90 pg/ml vs 351 ± 164 pg/ml, p = 0.004) and higher MMP-2 levels (4186 ± 1658 pg/ml vs 2303 ± 1059 pg/ml, p = 0.01) than control eyes. There was a significant trend towards lower VEGF-A (p trend = 0.001) and higher MMP-2 levels (p trend = 0.004) in eyes with MMD. After adjusting for age and gender, VEGF-A remained significantly lower and MMP-2 was significantly higher in eyes with MMD (p = 0.007 and p = 0.02 respectively). VEGF-A levels were also significantly lower in high myopes without MMD compared to controls (p = 0.004).

**Table 2:** Comparison of aqueous levels of cytokines in non-myopes and high myopes with and without myopic macular degeneration.

Cytokines, pg/ml	Controls	High myopes without myopic macular degeneration	High myopes with myopic macular degeneration	*P*-trend**	*P*-value^††^	Adjusted p (age, gender)
n	14	12	12			
Amphiregulin (% detectable)	28.6%	42.9%	20.0%	0.71	0.62	0.74
Amphiregulin*, pg/ml	1.08 ± 1.80	1.55 ± 1.93	0.69 ± 1.46	0.64	0.72	0.16
Pigment epithelium derived factor (PEDF), pg/ml	9816 ± 309	6723 ± 3711	8957 ± 4012	0.63	0.05	0.73
Matrix metalloproteinase 2 (MMP-2), pg/ml	2303 ± 1059	3045 ± 1667	4186 ± 1658	0.004	0.01†	0.02
Tissue inhibitor of metalloproteinase (TIMP-2), pg/ml	6509 ± 1222	5549 ± 2671	5237 ± 1988	0.27	0.30	0.17
Vascular endothelial growth factor isoform A (VEGF A), pg/ml	351 ± 164	172 ± 119	136 ± 90	0.001	0.0003‡	0.007
Interleukin 8 (IL-8), pg/ml	5.84 ± 2.89	5.59 ± 5.01	4.89 ± 3.32	0.43	0.81	0.38
Interleukin 6 (IL-6), pg/ml	4.46 ± 4.45	3.66 ± 3.69	3.39 ± 2.32	0.71	0.74	0.82
C-reactive protein (CRP), pg/ml	16906 ± 25608	2506 ± 3133	3520 ± 6200	0.07	0.05	0.12
Angiopoietin 2 (Ang2), pg/ml	52.7 ± 27.1	34.6 ± 20.0	40.3 ± 19.8	0.23	0.13	0.83

*19 eyes had amphiregulin levels below the limit of detection and these were considered to have zero concentration.

^†^Pairwise comparison with Bonferroni correction: controls vs high myopes without myopic macular degeneration (MMD), p = 0.83; controls vs high myopes with MMD, p = 0.01; high myopes without MMD vs high myopes with MMD, p = 0.35.

^‡^Pairwise comparison with Bonferroni correction: controls vs high myopes without MMD, p = 0.004; controls vs high myopes with MMD, p = 0.001; high myopes without MMD vs high myopes with MMD, p = 1.0.

**P-trend was analyzed across the ordinal categories of controls, high myopia without MMD and high myopia without MMD. A two-tailed *P*-value of <0.05 was considered statistically significant.

^††^P-value was obtained using one way analysis of covariance (ANOVA) across the 3 groups. A two-tailed *P*-value of <0.05 was considered statistically significant.

**Figure 1:**
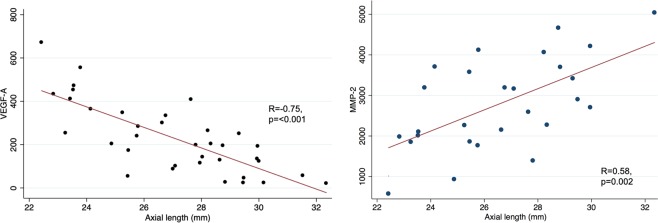
Scatterplots of concentrations of vascular endothelial growth factor isoform A (VEGF-A) and matrix metalloproteinase-2 (MMP-2) in aqueous humour samples, plotted against axial length.

The concentration of MMP-2 increased significantly with axial length (r = 0.58, p = 0.002), while the concentration of VEGF-A decreased significantly with longer axial length (r = −0.75, p < 0.001) (Fig. 1). Both MMP-2 and TIMP-2 were significantly associated with CT (r = −0.52, p = 0.03 and r = 0.54, p = 0.01) while VEGF-A levels were not significantly corelated (r = 0.35, p = 0.10, Table 3). In bivariate analysis the VEGF-A concentration was not significantly correlated with the MMP-2 concentration (r = −0.35, p = 0.07). After adding axial length to the model, both, VEGF-A concentration and the MMP-2 concentration remained to be significantly associated only with axial length, while they were no longer significantly associated with each other (*P* = 0.86). In bivariate analysis, a higher MMP-2 concentration was associated with a higher IL-6 concentration (*P* = 0.007; r: 0.50). After adding axial length to the statistical analysis, the association between the MMP-2 concentration and the IL-6 concentration was no longer statistically significant (*P* = 0.08). In bivariate analysis, a higher VEGF-A concentration was associated with a lower amphiregulin concentration (*P* = 0.03; r: −0.39). After adjusting for axial length, a lower VEGF-A concentration was associated with longer axial length (*P* < 0.001; beta: −0.68; B: −43.1; 95%CI: −59.5, −26.7) and with lower amphiregulin concentration (*P* = 0.043; beta: −0.27; B: −25.0; 95%CI: −49.2, −0.90). In the total study group, the amphiregulin concentration was not significantly associated with axial length (*P* = 0.42) in bivariate analysis.

**Table 3:** Correlation coefficients between aqueous cytokine levels, axial length and choroidal thickness.

Cytokine (pg/ml)	Axial Length	*P*-Value	Choroidal thickness	*P*-Value
	r		r	
Amphiregulin	−0.16	0.42	0.32	0.13
Pigment epithelium derived factor (PEDF)	−0.25	0.14	−0.08	0.74
Matrix metalloproteinase 2 (MMP-2)	0.58	0.002	−0.52	0.03
Tissue inhibitor of metalloproteinase (TIMP-2)	−0.18	0.34	0.54	0.01
Vascular endothelial growth factor isoform A (VEGF A)	−0.75	<0.001	0.35	0.10
Interleukin 8 (IL-8)	−0.07	0.68	0.04	0.85
Interleukin 6 (IL-6)	0.01	0.95	0.03	0.90
C-reactive protein (CRP)	−0.32	0.05	0.34	0.10
Angiopoietin 2 (Ang2)	−0.22	0.20	0.03	0.91

Axial length increased significantly (*P* < 0.001; beta: 0.27, B: 0.85; 95%CI: 0.21, 0.33) from the control group to the highly myopic group without MMD and finally to the highly myopic group with MMD. Adding the concentrations of any of the molecular factors to the model revealed no statistically significant associations between the molecular factor concentration and the presence of MMD. It held true in particular for the concentrations of VEGF-A (*P* = 0.25) and MMP-2 (*P* = 0.69).

## Discussion

In this study, we assessed the intraocular concentration of molecular factors potentially related to the pathogenesis of MMD, some of which have not been previously studied in eyes with MMD. We found that VEGF-A concentration in aqueous humour linearly decreased and the MMP-2 concentration linearly increased with longer axial length, statistically independently of MMD. The intraocular concentrations of all other molecular factors were not significantly correlated with axial length. MMD was strongly correlated with longer axial length, but not with the concentration of VEGF-A or MMP-2 or of any other molecular factor tested.

While the AL of the eye generally reaches adult length by the age of 13 years in most individuals^22,23^, it has been demonstrated that AL continues to increase significantly in myopic individuals even in the fourth decade^24^. The results of the current study may provide further insights into the molecular pathways responsible for this dysregulated and continued axial length elongation observed in highly myopic adults. MMPs are a group of enzymes that degrade extracellular matrix (ECM) components, regulated by inhibitors known as TIMPs. Elevated levels of MMP-2 have been found in the aqueous humour^16,17^ and vitreous humor of highly myopic eyes^7^, and our findings corroborate with these previous studies. MMP-2 has been shown to be present in Bruch’s membrane^25^, and is one of the metalloproteinases involved in the degradation of type IV collagen present in both the basement membranes of the RPE and choriocapillaris^26^. MMP-2 may thus be responsible for the weakening of the structural integrity of Bruch’s membrane. We speculate that high levels of MMP-2 could be involved in the etiology of axial elongation by mediating abnormal extracellular matrix breakdown in Bruch’s membrane^27^. Together with the mechanical stress on the RPE-Bruch’s membrane-choroid complex induced by axial elongation, weakening of Bruch’s membrane by MMP-2 could lead to the lacquer cracks and Bruch´s membrane defects commonly seen in association with marked axial elongation. The negative correlation of MMP-2, and corresponding positive correlation with TIMP-2, with choroidal thickness seen in our study suggests that this postulated degradation of Bruch’s membrane may also be related to the progressive thinning of the choroid seen in eyes with high myopia and MMD. The significant correlations of choroidal thickness with MMP-2 and TIMP-2, but not with VEGF-A further suggests that the mechanisms resulting in thinning of choroid observed in high myopia may be related to mechanical factors rather than ischemic factors.

Alternatively, breakdown of the extracellular matrix in the sclera by MMPs can cause weakening of the scleral wall with subsequent axial elongation^15^, and the elevation of MMP-2 as well as the manifestation of MMD may simply be a reflection of the processes driving axial elongation.

VEGF, PEDF and angiopoietin are growth factors produced by the retinal pigment epithelium (RPE) and are involved in the regulation of angiogenesis in the eye^28^. Several studies have measured lower concentrations of VEGF in the aqueous humor^18,29,30^ and a lower VEGF/PEDF ratio has been observed in aqueous humor samples of highly myopic eyes compared to controls^28^. The lower VEGF levels in highly myopic eyes have been suggested to be due to a dilution effect from the larger intraocular volume of these eyes^8,18,30^. Other reasons, besides a larger volume leading to dilution, for the lower VEGF concentration in axially long eyes may have been differences in the consistency of the vitreous body, which is less viscous in axially elongated eyes. A higher fluidity of the vitreous body may be associated with a faster turnover of VEGF out of the eye. We found a strong negative correlation of VEGF-A with axial length, but the dilution effect cannot explain the lack of correlation between the levels of other molecular factors with axial length. Similarly, Zhu *et al*. did not find a correlation between the aqueous concentrations of various inflammatory cytokines with axial length^18^. Alternatively, lower VEGF levels may reflect decreased production or increased internalization from the RPE in highly myopic eyes with MMD. VEGF has been shown to be essential for the maintenance of the choriocapillaris^14^. Low levels of VEGF may be related to the choroidal thinning and decreased choroidal vascularity seen in MMD. Evidence regarding the mechanisms behind choroidal thinning in myopia has been conflicting. Guthoff *et al*. measured axial length and thickness of the posterior ocular coats ultrasonographically, including the choroid, in 159 eyes with a spherical correction varying from +14.0 to −27.0 diopters^31^. They found a constant tissue volume regardless of the difference in axial length between hyperopic and myopic eyes, suggesting that choroidal thinning in myopia was a function of axial elongation and passive stretch. On the contrary, Read *et al*. measured axial length with optical biometry and choroidal thickness using optical coherence tomography in 104 children and found that the degree of choroidal thinning was substantially greater than that predicted by passive stretching of ocular tissue alone^32^. This finding implies that additional mechanisms, such as the low VEGF-A intraocular environment, may be involved in choroidal thinning in high myopes. Indirect evidence from studies on choroidal thickness after intravitreal injections of anti-VEGF agents suggest that a low intraocular VEGF environment leads to thinning of the choroid, but whether low VEGF levels and subsequent choroidal thinning is directly related to the development of MMD has remained unclear^33–36^.

However, the lack of correlation between choroidal thickness and VEGF-A levels in our study, in contrast to the strong correlation with axial length, suggests that axial elongation is the predominant factor driving low intraocular VEGF-A levels in these eyes. It may also suggest that the axial elongation-associated decrease in the VEGF-A concentration was not due to a reduction of VEGF production from a thinned/atrophied choroid. Longitudinal studies may be warranted to further assess the role of VEGF in the development of MMD. The decreasing intraocular concentration of VEGF-A with longer axial length may explain the protective role of axial myopia against age-related macular degeneration and diabetic retinopathy as shown in previous population-based studies^37–40^.

Myopia and its progression has been associated with both systemic and ocular inflammation^11,12,41^. Chronic inflammation presumably results in a weakening of scleral connective tissue with subsequent axial elongation^41^. We found no difference in the intraocular concentrations of pro-inflammatory cytokines between highly myopic eyes and non-highly myopic eyes in our study population, similar to the findings of Zhu and colleagues who studied the intracameral levels of Interleukin 1β, 6, 8, 10, 12p and did not find a significant association with axial length or refractive error^18^. Another group found no significant difference in the concentrations of IL-6 and IL-8 between highly myopic eyes and non-highly myopic eyes, although these authors found higher levels of interleukin-1 receptor antagonist (IL-1ra) and monocyte chemoattractant protein-1 (MCP-1)^42^. However, this does not exclude the possibility that previous inflammation might have contributed to the development of myopia in these patients, who by the time of examination had entered a quiescent non-inflamed state.

Overall, we did not find a molecular factor the intraocular concentration of which were clearly associated with the presence of non-neovascular MMD. This observation stems from the fact that none of these molecular factors, including VEGF-A and MMP-2, showed a step-wise difference between highly myopic eyes with MMD compared to highly myopic eyes without MMD. Rather, the decrease in VEGF-A concentration and increase in MMP-2 concentration showed a correlation with longer axial length. Adding the degree of MMD to the model did not reveal a significant correlation between the molecular factor concentrations and the MMD degree. Interestingly, Ohsugi *et al*. found that axial length increase was greater in highly myopic eyes compared with non-highly myopic eyes, and among the highly myopic eyes, axial length increase was greatest in those with myopic choroidal neovascularization^43^. These observations thus support the hypothesis that development of MMD may mainly reflect the severity of axial elongation and less likely to be a direct consequence of the specific molecular mechanisms that we studied. Our findings also suggest a possible role of MMP-2 in driving axial elongation and choroidal thinning in adulthood. This will require further evaluation in future longitudinal studies.

Limitations of our study should be discussed. First, our sample size was small, and the lack of sufficient statistical power may have prevented a statistical significance between the intraocular concentrations of the molecular factors examined and the presence of MMD or axial length. We performed a power calculation based on previous literature on VEGF-A levels, but it was not possible to do individual power calculations for all the molecular factors analyzed in this study. Second, the cross-sectional study design precluded the assessment of temporal relationships between the molecular factor levels and axial length and the presence of MMD. Third, in 19 eyes, the intraocular amphiregulin concentration were below the level of detection and were considered to be zero. Fourth, the study included the eyes of elderly cataract patients in whom the process of axial elongation had presumably ended. However, several studies have demonstrated that axial elongation of the globe does not cease in adolescence in highly myopic eyes, but continue to increase in adulthood^22–24,44–46^. This phenomenon is not well understood, but presents an opportunity for the molecular mechanisms underlying axial elongation to be studied via the assessment of ocular samples from adults. Fifth, our study was limited to eyes with MMD without myopic CNV. Wakabayashi observed a relatively higher aqueous VEGF concentration in highly myopic eyes with active CNV compared to highly myopic eyes without CNV, although both groups had lower VEGF levels compared to controls^29^.

In conclusion, the prevalence of non-neovascular MMD was not directly related to the intraocular concentrations of any molecular factor tested. Concentration of VEGF-A decreased and of MMP-2 increased with axial length, independently of the presence of non-neovascular MMD. These findings suggest that the predominant mechanism underlying the development of non-neovascular MMD may be axial elongation, driven in part by MMP-2 related mechanisms.

## Data Availability

The datasets generated during and/or analyzed during the current study are available from the corresponding author on reasonable request.
